# Unleash the dogs of death: metacaspase 5, microtubules, and hypersensitive response

**DOI:** 10.1007/s00299-025-03567-x

**Published:** 2025-07-30

**Authors:** Xin Zhu, Kunxi Zhang, Peijie Gong, Michael Riemann, Peter Nick

**Affiliations:** 1 Molecular Cell Biology, Joseph Gottlieb Kölreuter Institute for Plant Sciences, Karlsruhe Institute of Technology, Fritz-Haber-Weg 4, 76131 Karlsruhe, Germany; 2 College of Horticulture, Henan Agricultural University, Zhengzhou, 450000 Henan China; 3 Jiangsu Key Laboratory for Food Quality and Safety-State Key Laboratory Cultivation Base of Ministry of Science and Technology, Institute of Plant Protection, Jiangsu Academy of Agricultural Sciences, Nanjing, 210014 Jiangsu China

**Keywords:** *Cis*-3-hexenal, Hypersensitive response, Metacaspase, Programmed cell death, Tobacco BY-2, *Vitis rupestris*

## Abstract

**Key message:**

*Vitis* *rupestris* metacaspase 5, tethered to microtubules, drives grapevine. Hypersensitive response via calcium-dependent auto-processing, linking cytoskeletal dynamics to defence activation by elicitors.

**Abstract:**

Metacaspase 5 is a key player for the hypersensitive response of grapevine against biotrophic pathogens and must be activated rapidly as to prevent colonisation. This activation is likely to occur through changes in protein activity. By expressing a GFP fusion of metacaspase 5 from *Vitis rupestris* in tobacco BY-2 cells, we can show that this protein is bound to microtubules and that the overexpressors are more responsive to the cell-death-inducing elicitors, *cis*-3-hexenal and harpin. The disruption of microtubules and actin filaments by these elicitors can be blocked by inhibitors of dynamic turnover and stabilisation. Stabilisation of microtubules by taxol can mitigate *cis*-3-hexenal induced mortality. Mutations of the catalytic or the putative microtubule-binding sites of metacaspase 5 can suppress auto-processing of this enzyme in biochemical assay. Likewise, the response to *cis*-3-hexenal (cell death, induction of salicylate-related gene expression) is suppressed in cells, whilst the cytoplasmic remodelling is retained. Calcium and the sites for catalysis or microtubule binding are required for both auto-processing and enzyme activity. We arrive at a model, where metacaspase 5 is inactive when tethered to microtubules, but becomes unleashed for auto-processing upon defence-mediated microtubule breakdown.

**Supplementary Information:**

The online version contains supplementary material available at 10.1007/s00299-025-03567-x.

## Introduction

Plant immunity differs fundamentally from the induced immunity of vertebrates (Jones and Dangl 2006). It is of exclusively innate nature using receptors recognising molecules that are either originating from the pathogen itself or from the damage it inflicts upon the plant cell. There are two tiers of immunity, differing with respect to spatial distribution. As a first response, the invaded cell deploys a local defence triggered by membrane-located receptors. This local response includes warding off invasive pathogen structures, for instance by callose deposition; producing defence compounds, so-called phytoalexins, for instance the grapevine stilbenes; and synthesizing pathogenesis-related proteins, for instance chitinase (Somsich and Hahlbrock 1998). When this local response is not sufficient, or when the attack is undertaken by a biotrophic pathogen that by co-evolution has acquired effectors able to suppress this initial basal immunity, the attacked cell can launch a second tier of immune responses, whereby it commits suicide, thus killing the invader, but protecting the neighbouring cells. This so-called hypersensitive response (HR) is again controlled by specific host receptors, this time located in the cytoplasm, that can recognise the effectors secreted by biotrophic pathogens to quell the initial basal immunity. The two tiers of immunity differ in terms of specificity as well. The initial basal defence is triggered by pathogen-related molecular patterns (PAMPs) that are generic for an entire group of pathogens (Jones and Dangl 2006). A receptor binding a conserved peptide structure in flagellin allows recognising the majority of bacteria, or a receptor for chitin enables to detect any fungus trying to attack. In contrast, the second tier is far more specific because there exist multiple targets by which effectors secreted by biotrophic pathogens can hijack basal immunity. Therefore, also the host receptors detecting those effectors encoded by so-called R genes (for Resistance), are far more diverse and, hence, specific, than the generic PAMP receptors.

The signal-transduction chains underlying the two tiers of immunity are partially overlapping, but also show some bifurcations. This has been dissected using a grapevine cell system, where basal defence can be triggered by flg22, a conserved peptide motif in bacterial flagellins, whereas a cell death-related defence can be triggered by harpin, an elicitor from the phytopathogenic bacterium *Erwinia amylovora* (Chang and Nick 2012). Whilst both elicitors trigger an influx of calcium, an apoplastic oxidative burst deployed by the membrane-located NADPH oxidase respiratory burst oxidase Homologue, a MAPK cascade, and the induction of transcripts for genes involved in stilbene phytoalexins, the two defence responses do differ with respect to the temporal sequence of these events. For flg22-triggered defence, the calcium influx is swift, and the oxidative burst follows later. For harpin-triggered defence, this sequence is inverted. This altered temporal signature culminates in differences of the stilbene output in response to flg22, the inactive glycoside α-piceid accumulates, whilst harpin triggers the accumulation of the aglycon resveratrol and its oxidative oligomers, the viniferins. These aglycones are highly potent against pathogens, but also toxic to the producing cell, which might be the reason that in case of basal immunity (elicited by flg22), they are glycosylated to be sequestered in the vacuole (Chang et al. 2011).

During the search for molecular events reflecting this bifurcation of defence signalling, we have identified oxylipin metabolism as possible branching point. Oxylipins are formed in response to various abiotic and biotic stresses, whereby unsaturated fatty acids, released from the plastid membrane, are oxidised by lipoxygenases culminating in jasmonates, crucial regulators of the plant stress response (for a comprehensive review see Wasternack and Song 2017). A central product of the initial lipoxygenation, 13-hydroperoxy octadecatrienoic acid, is converted further by allene oxide synthase, a cytochrome P_450_ enzyme belonging to the CYP74 family. However, a member of the same family, hydroperoxide lyase, can convert the same substrate into the volatile aldehydes *cis*-3-hexenal and 2-*trans*-hexenal (Akaberi et al. 2018). One of these isomers, *cis*-3-hexenal, can induce cell death, whilst 2-*trans*-hexenal fails to do so, indicating that this biological response depends on a stereo-isomeric-binding site. The concurrent pathway leading to jasmonates does not result in cell death, as shown by a comparative study in grapevine cells, where induction of basal immunity by flg22 led to accumulation of the bio-active conjugate JA-Ile, which was not accompanied by any increase of mortality, whilst induction of cell death-related immunity by harpin failed to induce any significant accumulation of jasmonates (Chang et al. 2017). However, basal and cell death-related defence are not only concurrent with respect to the bifurcation of oxylipin synthesis for jasmonates versus *cis*-3-hexenal, and with respect to their activation during alternative defence contexts. Both defence types can also seem to be functionally antagonistic because the cell death triggered by harpin can be suppressed by exogenous jasmonic acid (Akaberi et al. 2018).

The molecular basis of this functional antagonism is far from understood, but there are indications that it is linked with the cytoskeleton. Actin remodelling is a hallmark of programmed cell death (PCD) in general, and of HR in particular (for a comparative review over different eukaryotic life forms, see Franklin-Tong and Gourlay 2008). Conversely, for grapevine cells, the elicitation of cell death-related defence by the elicitor harpin is heralded by a massive actin remodelling (Chang et al. 2015), whilst flg22 produces only a very mild response (Guan et al. 2013). When actin remodelling is mitigated by adding exogenous auxins, or when actin filaments are eliminated by Latrunculin B, the cell death response is suppressed, demonstrating that actin remodelling is necessary to execute cell death. It is not sufficient though, for instance, aluminium ions can phenocopy harpin with respect to actin remodelling but do not evoke a subsequent cell-death response (Wang et al. 2022).

Whilst HR, at first sight, resembles animal apoptosis, the cellular and molecular details differ, such that one has to see HR rather as convergent, not as homologous phenomenon (for review see Jones 2001). For instance, plants lack caspases, the central executors of apoptosis. Instead, they use metacaspases, proteases that do not share sequence similarity with exception of the functionally relevant peptide motives in the active centre (for review see del Pozo and Lam 1998). In grapevine, for instance, they have been shown to participate in ovule abortion in seedless grapes (Zhang et al. 2013). Metacaspases come in two types that differ with respect to their function and are conserved from the Chlorophytes onward. The type-I metacaspases (comprising the VrMC2) are activated by calcium and then break down proteins in a constitutive way (van Midden et al. 2021). For HR in *Arabidopsis*, genetic ablation of the I metacaspase AtMC1 almost completely eliminated HR, meaning that this type-I metacaspases act as an executor (Coll et al. 2010). The type-II metacaspases rather play a regulatory role. Using the HR of grapevine in response to infection with the biotrophic pathogen *Plasmopara viticola* as paradigm, we could show that the type-I metacaspase MC2 and the type-II metacaspase MC5 from the North American wild grapevine *Vitis rupestris* were specifically upregulated during HR mediated by the R-locus *Resistance to Plasmopara viticola 3* (Gong et al. 2019). The two metacaspases were found in different sites of the cell, MC2 in the ER, MC5 in the cytoplasm and the nucleus. Also, transcriptional regulation differs (Gong et al. 2023): Transcripts for MC5 are only induced by harpin, MC2 also by methyl jasmonate (Gong et al. 2023).

Type-II metacaspases show auto-processing upon calcium activation (Watanabe and Lam 2011).

Recently, the crystal structure for the type-II metacaspases AtMC4 could be solved (Zhu et al. 2020), revealing a conserved cysteine at position 139 as well as a conserved lysine at position 226 as crucial for auto-processing. Thus, a rapid auto-cleavage of the type-II grapevine metacaspase VrMC5 is predicted as early or even first committed step of HR. We test this implication in vivo using ectopic expression in tobacco BY-2 cells and in vitro using recombinantly expressed proteins. In addition to the wild-type VrMC5, we use engineered variants, where catalytic sites, or processing target sites have been mutated. As triggers for HR, we use *cis*-hexenal and the elicitor harpin.

## Materials and methods

### Plant cell materials

We conducted the experiments with variant tobacco BY-2 cell lines VrMC5-GFP, which exogenously expressing with metacaspase 5 from *Vitis rupestris* fused with C-terminal GFP. Two variants VrMC5-GFP were generated by site-directed mutagenesis from plasmid template pH7FWG2.0/ VrMC5 (Gong et al. 2019). The targets of the mutation were two sites, cysteine 139 replaced by an alanine and arginine 226 replaced by a glycine, inferred as crucial from investigations in the respective Arabidopsis homologues (Vercammen et al. 2004). These tailored variants were obtained by PCR using the oligonucleotide primer pairs 5’-TACGATAGTGTCGGATTCGGCCCACAGCGGTGGCCTGAT-3’ and 5’- ATCAGGCCACCGCTGTGGGCCGAATCCGACACTATCGTA-3’ for C139A, and the primer pairs 5’- GCGGCTATGTGAAGAGCGGATCTCTGCCGCTTTC-3’ and 5’- GAAAGCGGCAGAGATCCGCTCTTCACATAGCCGC-3’ for R226G. The PCR reaction programme consisted of a primary denaturation step at 98 °C for 3 min, followed by 17 cycles of denaturation at 98 °C for 10 s, annealing at 55 °C for 30 s, and elongation at 72 °C for 5 min. The PCR products were digested by 1 μL of Dpn I restriction enzyme (10 U/μL, NEB, Germany) at 37 °C for 3 h to eliminate the non-mutated parental plasmid. The constructions were then transformed into BY-2 cells based on an *Agrobacterium*-mediated transient transformation (Buschmann et al. 2011) with slight modifications for stable transformation (Klotz and Nick 2012). The transformants were selected with 60 mg/L hygromycin. Suspension cells of tobacco BY-2 were cultivated in a modified Murashige and Skoog (MS) medium as described by Schneider et al. (2015). The microtubule marker line TuA3 (Kumagai et al. 2001) was cultivated in the same medium supplemented with 25 mg/L Kanamycin, the actin marker line GF11 (Sano et al. 2005) as well as the lines overexpressing VrMC5-GFP and its variants in the same medium supplemented with 30 mg/L hygromycin. For each transformation, three independent clonal strains were maintained that were used as biological replicates.

### Stress and inhibitor treatments

Cells were treated at day 3 after sub-cultivation at the peak of mitotic activity. The samples for RNA extraction were collected at 30 min after addition of the respective compound, whilst cell mortality and proteins were assayed after 15 min. As triggers for PCD, we either used the small aldehyde *cis*-3-hexenal along with its inactive isomer *trans*-2-hexenal (Akaberi et al. 2018), or the bacterial elicitor harpin from the phytopathogenic bacterium *Erwinia amylovora* (Qiao et al. 2010). The cells were treated with either 12.5 μM *cis*-3-hexenal (from a 50% solution in triacetin) or *trans*-2-hexenal (from a 98% solution in triacetin). In some experiments, the two compounds were added following a pre-incubation for 1 h with either 10 μM of the actin polymerisation inhibitor Latrunculin B (Lat B) (stock in DMSO); 1 μM of the actin stabiliser phalloidin (stock in ethanol); 10 μM of the inhibitor of microtubule assembly oryzalin was used to treat TuA3 (stock in DMSO); or 10 μM of the microtubule stabiliser taxol (stock in DMSO) for 1 h. All the tests were set up with appropriate solvent controls to account for potential side effects of the solvent. These were 40 ppm of triacetin in case of *cis*-3-hexenal and *trans*-2-hexenal; 1% of DMSO in case of LatB, oryzalin, and taxol; and 0.1% of ethanol in case of phalloidin.

### Microscopic analysis

Mortality was determined using the Evans Blue Dye Exclusion assay (Gaff and Okong’O-Ogola 1971) with minor modifications (Kühn et al. 2013). Cells that have lost their membrane integrity are stained blue, whilst viable cells remained unstained. Data represent means and standard errors from three independent experimental series, scoring 500 individual cells per measurement. All fluorescent proteins were observed by spinning disc confocal microscopy as described in Akaberi et al. (2018), at excitation at 488 nm and emission at 509 nm. The TRITC signal from in situ staining of actin and microtubules was recorded at 610 nm upon excitation at 561 nm.

### Visualisation of microtubules and actin filaments

Microtubules were visualised by indirect immunofluorescence using a monoclonal antibody against α-tubulin (ATT, Sigma, Deisenhofen, Germany) diluted 1:500, and a secondary anti-mouse IgG antibody conjugated to tetramethylrhodamine (TRITC, Sigma, Deisenhofen, Germany) diluted 1:250 at day 3 after sub-cultivation following the protocol published by Eggenberger et al. (2007). Actin filaments were stained with TRITC-phalloidin after mild fixation and permeabilization as described previously Maisch and Nick (2007).

### Protein extraction and western blot analysis

To verify the expression pattern of MC5 and its variants in response to signals, cells were collected at days 0 (stationary phase) or 3 (mitotic phase) after sub-cultivation. Soluble proteins were extracted according to Jovanović et al. (2010) and analysed by western blotting using monoclonal mouse antibodies against the GFP reporter (anti-green fluorescent protein antibody, Sigma-Aldrich, Deisenhofen, Germany) in a dilution of 1:1000, and a goat polyclonal anti-mouse IgG conjugated to alkaline phosphatase (Sigma-Aldrich, Germany) in a dilution of 1:25000 for signal development. Tyrosinated and de-tyrosinated α-tubulin were fractionated based on their differential affinity with the anti-micro-tubular compound ethyl-*N*-phenylcarbamate (EPC) according to Wiesler et al. (2002).

### Recombinant expression of VrMC5 and its variants

The coding sequence of the *VrMC5* was inserted into the pET21b expression vector. The resulting construct encoding the VrMC5 protein fused with 6 × His tag was expressed in *E. coli* strain BL21(DE3) following heat shock transformation. The VrMC5 variants were generated by site-directed mutagenesis as described above. The entire volume (100 mL) of the pre-culture was inoculated into 3 L of LB medium complemented with 100 μg/mL of ampicillin and cultivated at 37 °C for 3–4 h. When the OD_600_ reached 0.8–1.0, 80 μM of isopropyl-*β*-d-thiogalactopyranoside (IPTG) was added. After incubation overnight at 18 °C, cells were harvested by centrifugation (10,000 *g*, 20 min, 4 °C, Sorvall LYNX 4000 Superspeed Centrifuge, Thermo Scientific, Germany). To extract the proteins, cells were resuspended in 200 mL ground buffer (50 mM Tris, 5 mM EDTA, 300 mM NaCl, 10%w/v glycerol, pH 7.8), spun down (10,000 *g*, 10 min, 4 °C), and resuspended in 60 mL ground buffer, prior to two passages through a French Press at 1000 bar. Cell debris was removed by centrifugation at 15,000 *g* for 30 min twice at 4 °C, and the cleared supernatant was precipitated at 4 °C overnight with 93% ammonium sulphate buffer (3.3 M ammonium sulphate, 50 mM Tris/HCl, pH adjusted to 7.8 with solid ammonium sulphate). Precipitated protein was collected by centrifugation (10,000 *g*, 30 min, 4 °C) and dissolved in 40 mL storage buffer (50 mM Tris, 300 mM NaCl, 10%w/v glycerol, pH 7.8). The His-tagged proteins were purified by affinity chromatography on a Ni–NTA agarose column. The concentrated recombinant protein was dissolved in 2 mL ground buffer and used for the analysis of enzymatic activity.

### Assay for metacaspase enzymatic activity

To measure metacaspases activity, a fluorometric assay for type-II metacaspases was used (Vercammen et al. 2004). The assays were carried out in a total volume of 100 μL reaction mixture containing 30 nM of purified recombinant protein, 100 μM of the fluorogenic caspase substrate Boc-GRR-Amino-Trifluoromethyl-Coumarin (Bachem) and reaction buffer (50 mM Tris, 100 mM NaCl, 5 mM DTT, 0–50 mM CaCl_2_, pH 7.5). Each assay was set up in triplicates in a 96-well plate and the release of Amino-Trifluoromethyl-Coumarin (AMC) was continuously monitored every minute for 30 min at 25 °C with a micro-titre plate reader (Synergy HT, BIO-TEK) at an excitation wavelength of 360 nm and an emission wavelength of 460 nm. Data were read out as increases in relative fluorescence as a function of time. The specific enzyme activity was calculated as nmol of substrate hydrolysed per mg of protein per min using a standard curve of AMT in the reaction buffer.

### Quantification of substrates by real-time qPCR

The suspension cells were collected by centrifugation (5 min, 10,000 *g*) and the total RNA extracted using the innuPREP RNA Mini Kit (Analytik Jena AG, Germany) following the producer manual. 1 μg of purified RNA was reverse-transcribed into cDNA using the M-MuLV cDNA Synthesis Kit (New England BioLabs; Frankfurt am Main, Germany) according to the instruction of the producer. The relative transcript abundance of the selected genes was measured by quantitative real-time PCR (qRT-PCR). Transcripts for *PALA* (*PHENYLAMMONIUM LYASE A*), *PALB* (*PHENYLAMMONIUM LYASE B*), *ICS1* (*ISOCHORISMATE SYNTHASE 1*), *PR1a* (*PATHOGENESIS-RELATED PROTEIN 1a*) and *EF1α* (*Elongation Factor 1α*) were amplified using the oligonucleotide primers given in Supplemental Table 1. The reactions were performed using a CFX96TM real-time PCR cycler (Bio-RAD, München). As housekeeping gene for normalisation, tobacco *EF1α* was used as an internal standard. The steady-state transcript levels were determined using the -DDC_t_ method by Livak and Schmittgen (2001) using the value of the untreated control as reference. The data represent mean and standard error from three independent biological replicates, each in technical triplicates.

## Results

### VrMC5 decorates nucleus-associated microtubule arrays

**Fig. 1:**
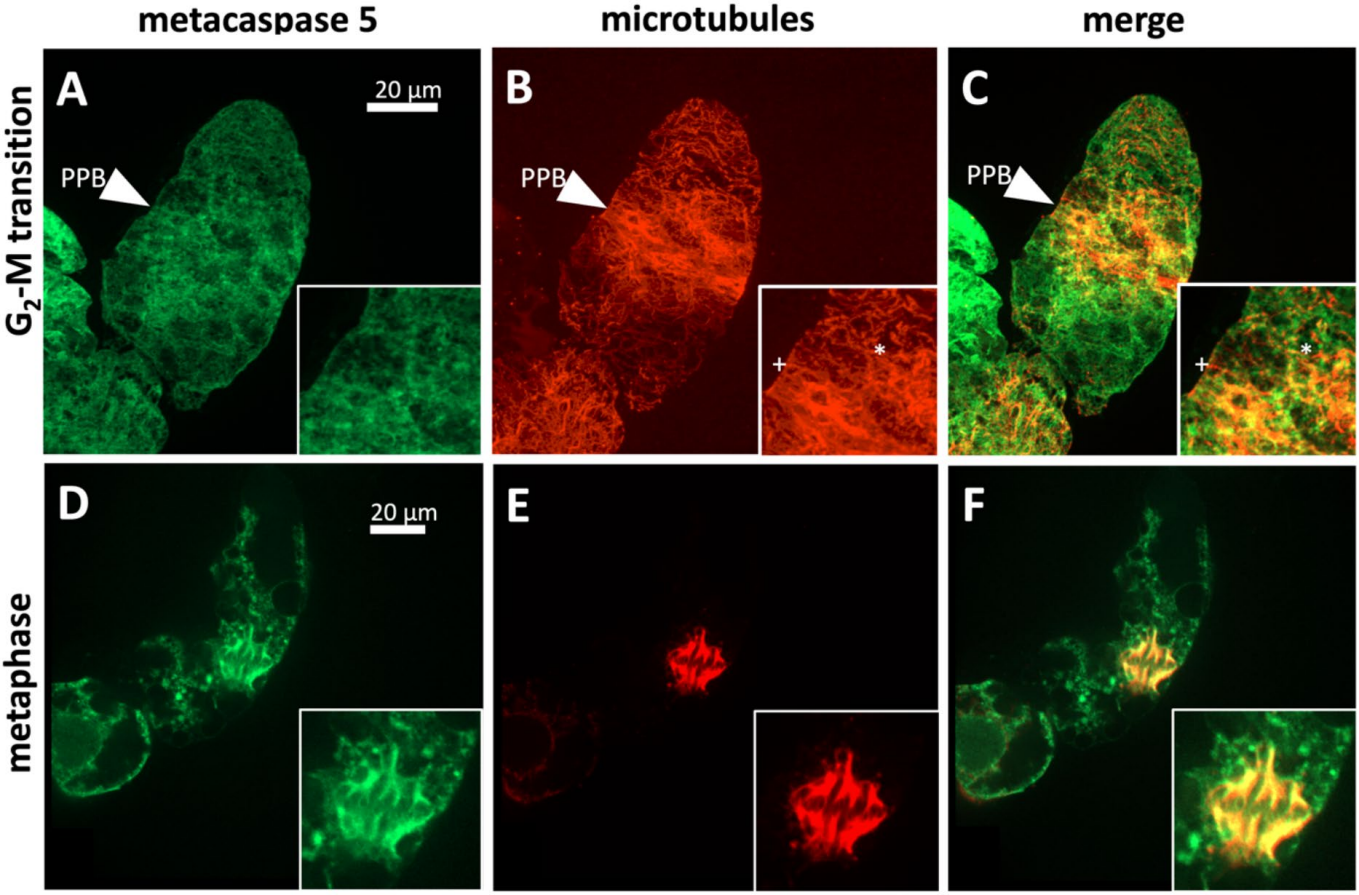
Subcellular localisation of *Vitis rupestris* metacaspase 5 (VrMC5) in tobacco BY-2 cells as hosts during G_2_/M transition and metaphase. Metacaspases are visualised by a fused GFP reporter, microtubules by immunofluorescence using a secondary antibody conjugated to tetramethylrhodamine. *PPB* preprophase band, + indicates microtubules not decorated by VrMC5, * indicates VrMC5 decorating a microtubule like beads on a string

**Fig. 2:**
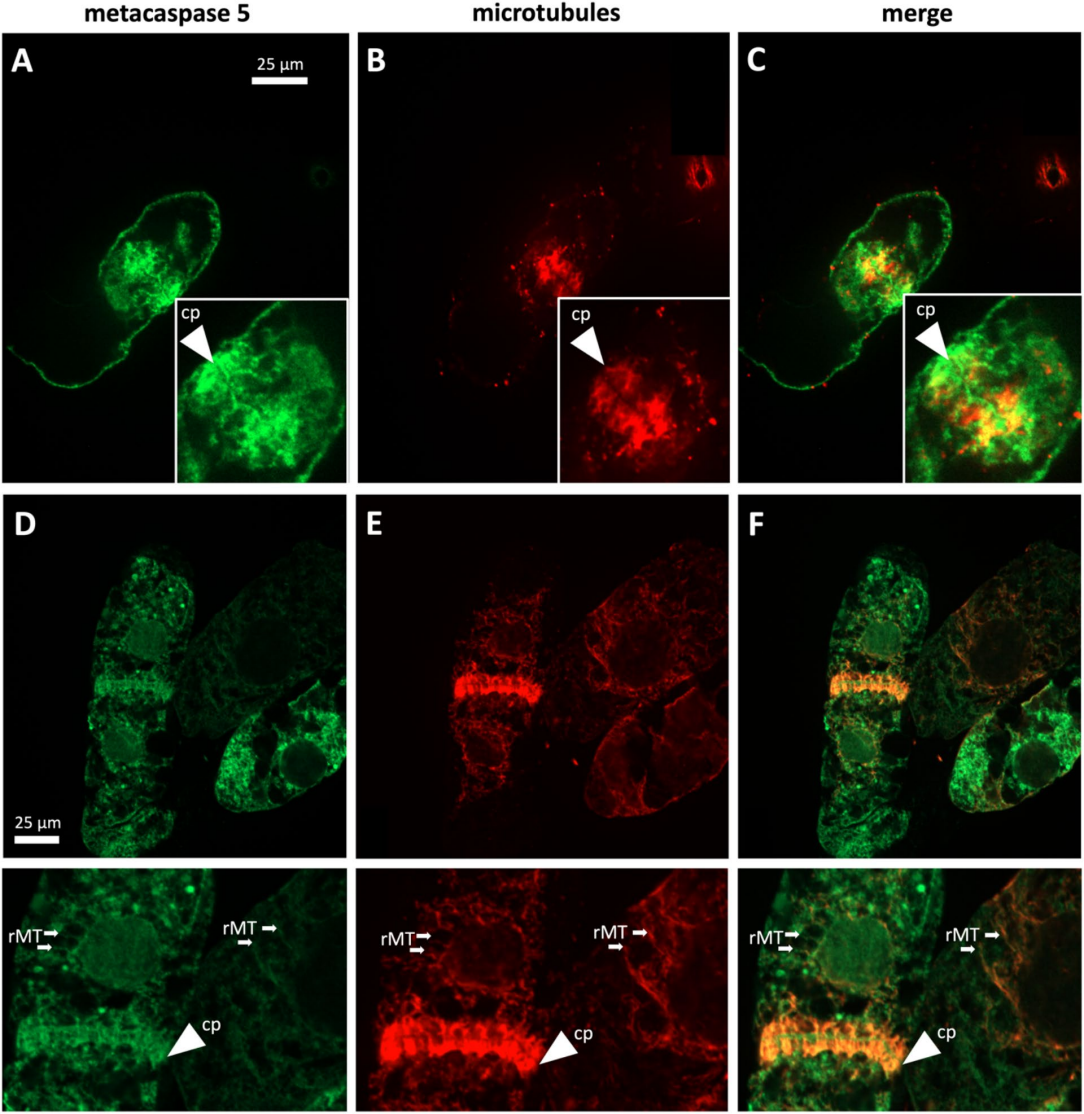
Subcellular localisation of *Vitis rupestris* metacaspase 5 (VrMC5) in tobacco BY-2 cells as hosts during telophase and cytokinesis. Metacaspases are visualised by a fused GFP reporter, microtubules by immunofluorescence using a secondary antibody conjugated to tetramethylrhodamine. *Cp* cell plate, *rMT* radial microtubules emanating from the nuclear envelope of the daughter nucleus

To get deeper insights into the function of VrMC5, subcellular localization was followed by a C-terminal fusion of VrMC5 with GFP in tobacco BY-2 suspension cells (Figs. 1, 2, Suppl. Fig. S1). A punctate signal in the cytoplasm was seen that partially was reminiscent of microtubules. Dual visualisation by immunofluorescence for α-tubulin by immunofluorescence revealed labelling with the preprophase band during the G_2_/M transition (Fig. 1A, B, C). During metaphase, the signal stained the spindle in a contiguous manner (Fig. 1D, E, F). During early telophase, VrMC5 was accumulating at the peripheral regions of the phragmoplast (Fig. 2A, B, C), whilst staining the mature phragmoplast evenly during cytokinesis (Fig. 2D, E, F). In addition, green-fluorescent speckles appeared in the cytoplasm of the daughter cells, but also at the newly formed nuclear envelopes of the daughter nuclei. During G_1_, the VrMC5-GFP signal was cytoplasmic and reticulate (Suppl. Fig. S1A) and did not show any relationship with the cortical microtubules (Suppl. Fig. S1B, C). However, confocal section at the nuclear plane revealed that the green signal co-localised with the radial microtubules that tether the nucleus (Suppl. Fig. S1D, E, F).

### VrMC5 is co-purified with α-tubulin by EPC-affinity chromatography

**Fig. 3:**
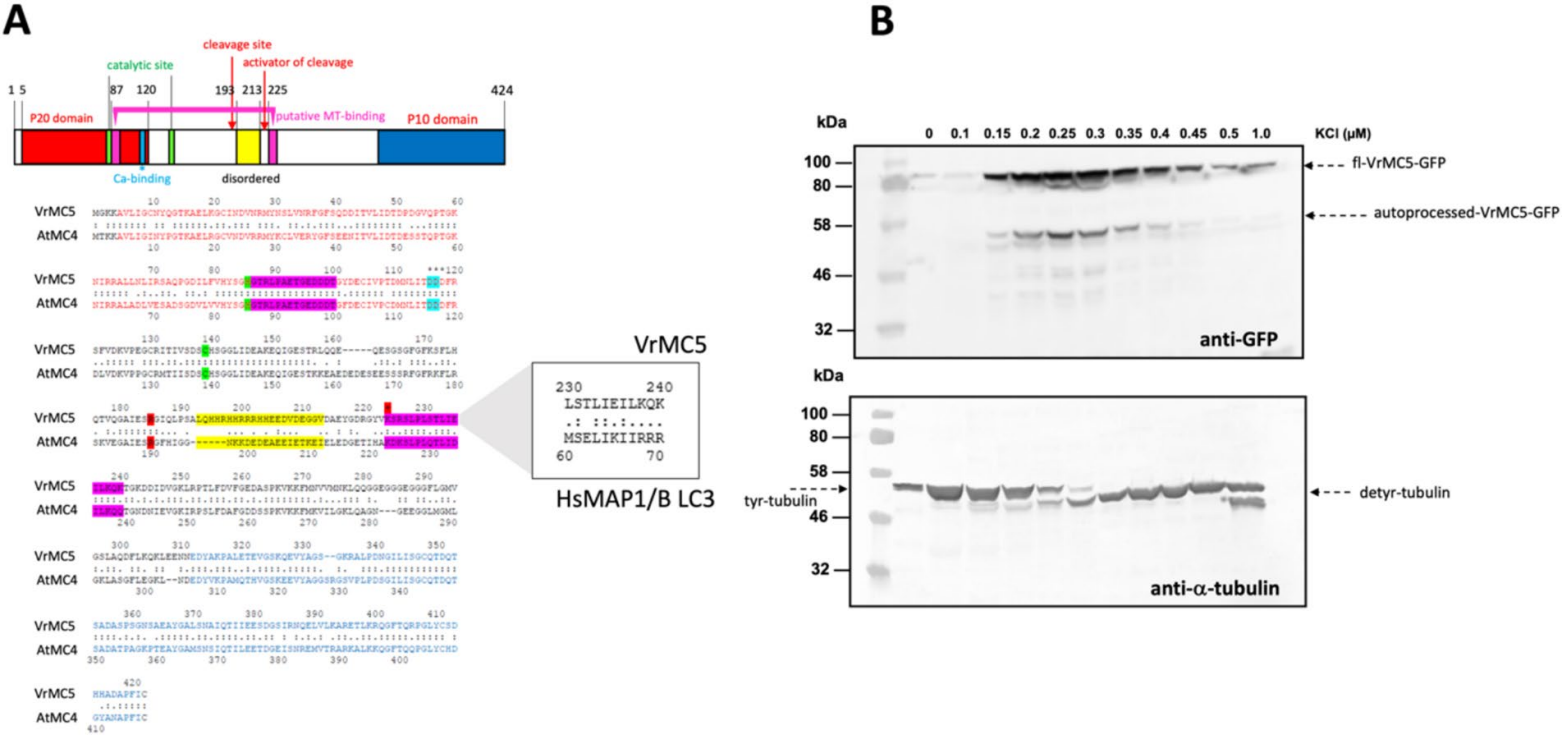
Biochemical properties of *Vitis rupestris* metacaspase 5 (VrMC5). **A** Domain structure of VrMC5 and alignment with the class-II metacaspases AtMC4 from *A. thaliana*. Colours highlight the respective domains. Inset shows the putative microtubule-binding domain and its similarity to the microtubule-binding domain of human MAP1B. **B** Co-elution of VrMC5-GFP with de-tyrosinated α-tubulin during chromatography based on the affinity for ethyl-*N*-phenylcarbamate (EPC), a tubulin-binding herbicide, using increasing ionic stringency for elution by increasing the concentration of KCl

To test whether the co-localization of VrMC5 with nucleus-associated microtubules is reflected by binding to tubulin, we used ethyl-*N*-phenyl carbamate (EPC) to separate tyrosinated and de-tyrosinated pools of α-tubulin (Wiesler et al. 2002). When we probed the profiles for the VrMC5-GFP fusion, we detected a band at around 80 kDa (close to the size of 73 kDa predicted for the fusion protein) which mostly co-eluted (Fig. 3A) with de-tyrosinated α-tubulin (Fig. 3B). A smaller band eluting with the same profile was matching well with the expected size of the auto-processed C-terminal VrMC5-GFP fragment carrying the GFP fusion.

### Overexpression of VrMC5 amplifies cell death in response to triggering signals

**Fig. 4:**
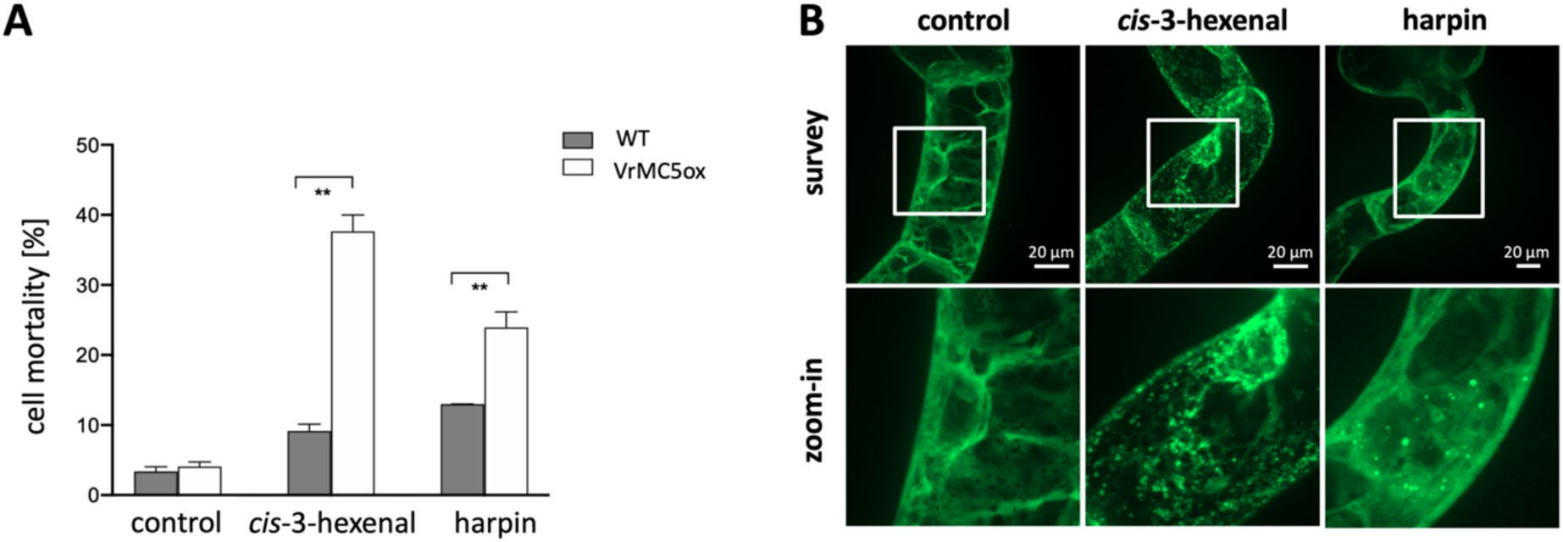
Overexpression of VrMC5-GFP enhances the response of tobacco BY-2 cells to inducers of programmed cell death. **A** Mortality in response to as compared to the solvent control (triacetin, 625 ppm, 15 min), *cis*-3-hexenal (12.5 μM, 15 min) and harpin (30 μg/mL, 48 h) in mitotic cells (3d after sub-cultivation). Data represent the mean ± standard error of four independent biological replicates, ** indicates significant difference value *P* < 0.01 (Student’s *t *test). **B** Response of subcellular localisation of VrMC5-GFP to the same treatments assessed at the same time points by spinning disc confocal microscopy

To get insights into the function of VrMC5, we triggered PCD either by *cis*-3-hexenal or harpin. Whilst *cis*-3-hexenal is produced by the plastidic hydroperoxyl lyase in response to stress and might act as a systemic signal orchestrating actin-dependent HR (Akaberi et al. 2018), harpin is a virulence factor of the phytopathogenic bacterium *Erwinia amylovora* inducing inappropriate HR in the host (Qiao et al. 2010). Both triggers induced elevated cell death in VrMC5-GFP over the wild type (Fig. 4A). For *cis*-3-hexenal mortality was amplified fourfold, for harpin two-fold. For *cis*-3-hexenal, VrMC5-GFP became disrupted into fluorescent dots (Fig. 4B), whilst harpin caused a breakdown of cytoplasmic strands such that a diffuse signal coexisted with a few dots and speckles.

### Cell death in response to *cis*-3-hexenal requires microtubular breakdown

**Fig. 5:**
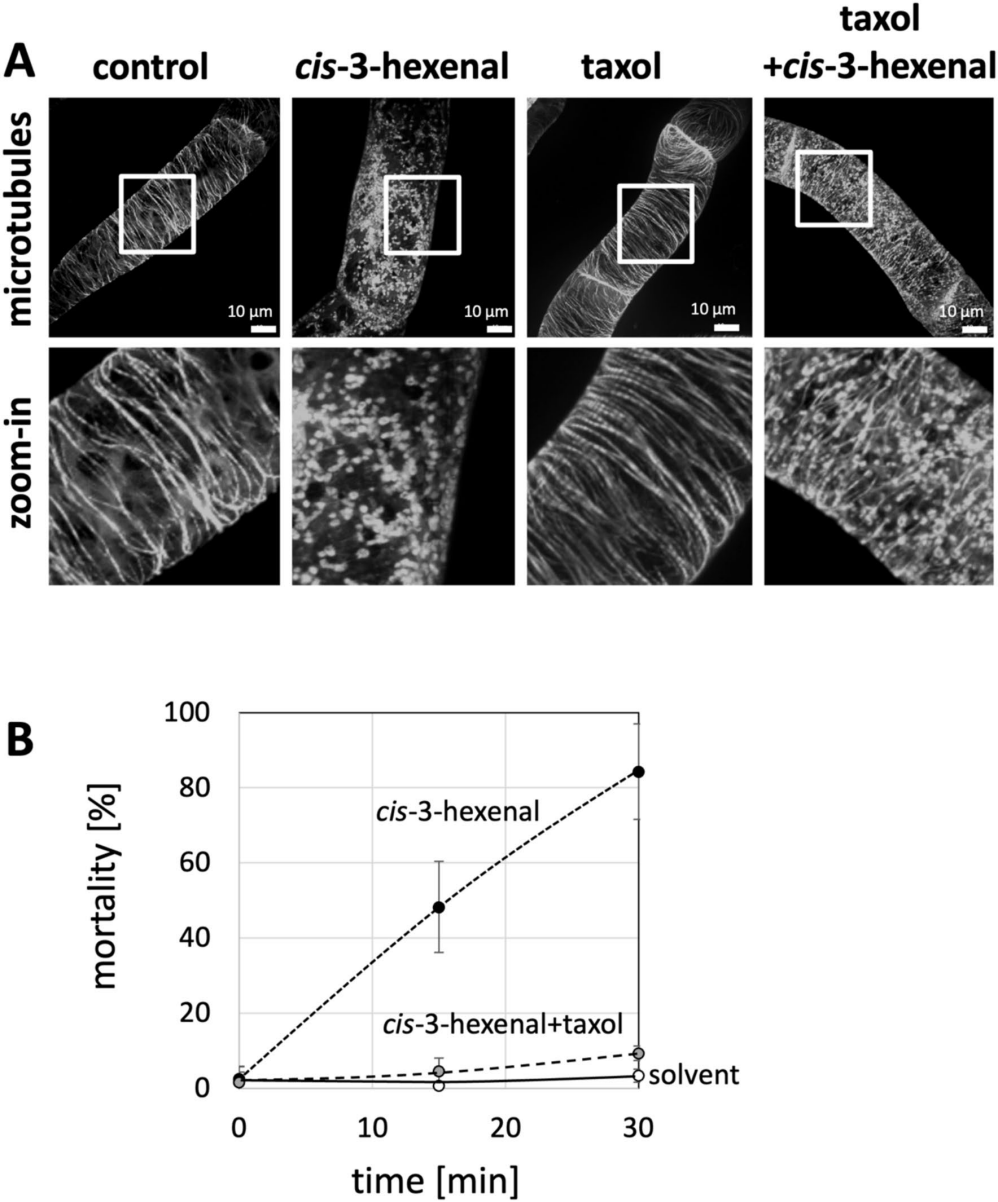
Microtubule breakdown and mortality in response to *cis*-3-hexenal can be suppressed by taxol. **A** Representative images of microtubules visualised by GFP-NtTUA3 are shown for the control treatment (triacetin, 625 ppm, 10 min), *cis*-3-hexenal (12.5 μM, 10 min), the stabilising compound taxol at 1 μM for 2 h, and the same taxol pre-treatment, but followed by treatment with *cis*-3-hexenal (12.5 μM, 10 min). Images show geometric projections of confocal z-stacks collected in the cortical region of the cell. **B** mortality induced by *cis*-3-hexenal without or with pre-treatment with taxol as compared to the solvent (triacetin, 625 ppm). Data represent means and standard errors from three independent experiments scoring 1500 individual cells per data point

We wondered whether cis-3-hexenal might act through disruption of microtubules, such that VrMC5-GFP is released and activates cell death. If so, *cis*-3-hexenal should disrupt microtubules and this disruption should precede the onset of cell death (observable from 15 min after application). Moreover, stabilisation of microtubules by taxol should significantly mitigate the mortality in response to *cis*-3-hexenal. In fact, *cis*-3-hexenal had already fully disrupted microtubules at 10 min (Fig. 5A). A pre-treatment with taxol at a concentration that was still not leading to bundling, suppressed microtubule disruption by *cis*-3-hexenal. The pre-treatment with taxol also suppressed the mortality in response to *cis*-3-hexenal (Fig. 5B).

Microtubules are intimately linked with actin filaments, which are also disrupted by *cis*-3-hexenal (Akaberi et al. 2018). Using the actin marker line FABD2-GFP, we observed that also actin filaments were rapidly disrupted (within 10 min) by *cis*-3-hexenal but could be partially rescued by pre-treatment with phalloidin (Suppl. Fig. S2).

### Cell death in response to *cis*-3-hexenal differs in auto-processing mutants of VrMC5

**Fig. 6:**
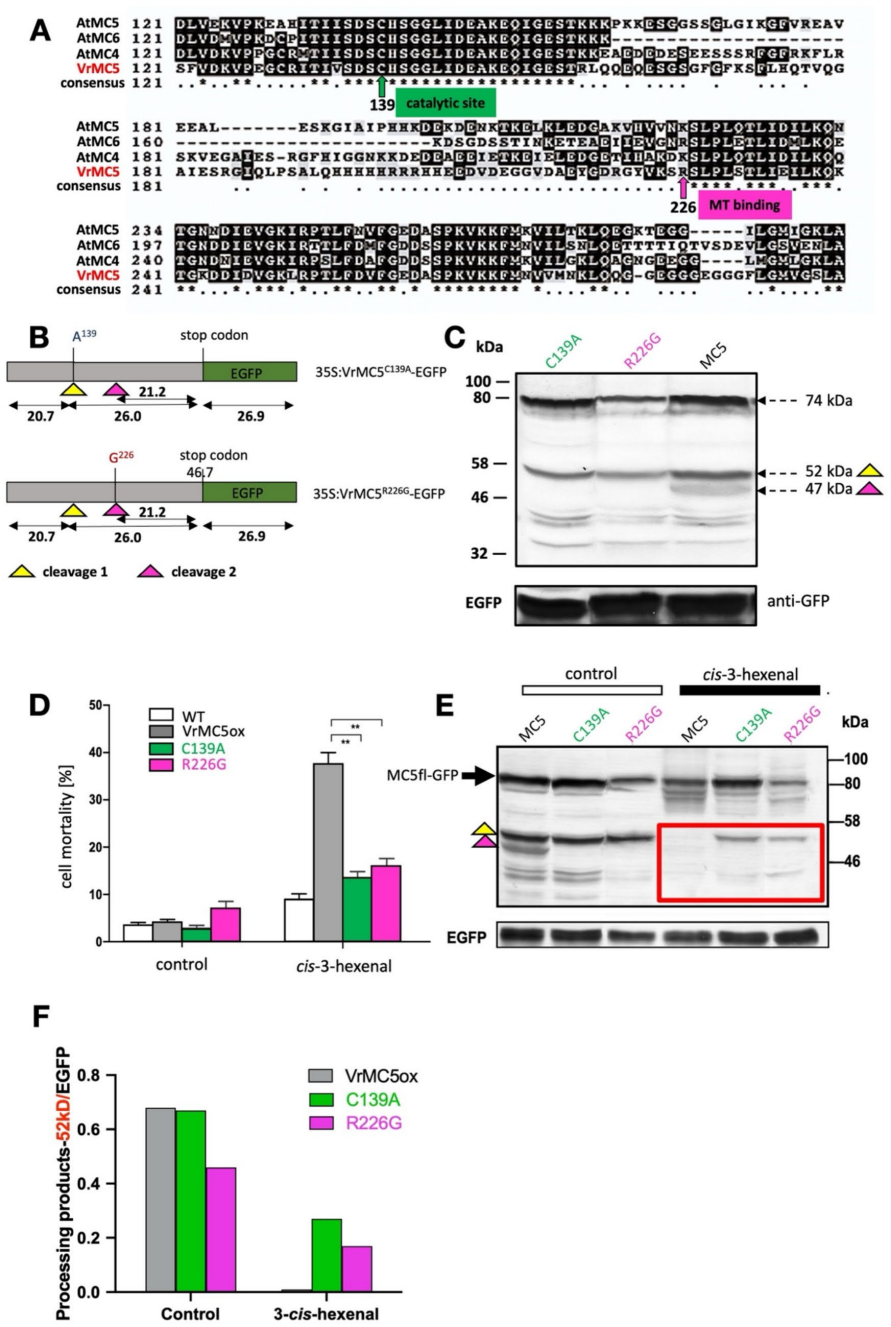
Engineering auto-processing of VrMC5-GFP. **A** Part of the alignment of VrMC5 with class-II metacaspases of *A. thaliana* to show the positions of the engineered sites. **B** Structure of the two processing mutants and predicted fragment sizes. **C** Auto-processing of VrMC5-GFP and the two mutants upon extraction from tobacco BY-2 cells expressing those constructs under control of a CaMV 35S promoter. Total proteins were extracted at day 3 after sub-cultivation and subjected to immunoblot analysis using an anti-GFP antibody. **D** Functional consequences of auto-processing mutants on cell mortality of *VrMC5-GFP* in response to *cis*-3-hexenal (12.5 μM, 15 min). Data represent the mean ± standard error (SE) of four independent biological replicates,** indicates significant differences at *P* < 0.01 (Student’s *t *test). **E** Modulation of self-processing in *VrMC5-GFP* variants in response to *cis*-3-hexenal (12.5 μM, 30 min) as compared to the untreated control probed by an anti-GFP antibody

To address whether self-cleavage is functionally relevant for the type-II metacaspases VrMC5 as well, we engineered two variants where two crucial amino acids were mutated (Fig. 6A), shown to be necessary for auto-processing of the homologous AtMC4 (Zhu et al. 2020). A highly conserved cysteine at position 139 was replaced by an alanine (C139A). Alternatively, the conserved arginine at position 226 (at the beginning of the putative microtubule-binding domain, Fig. 3A, inset) was replaced by a glycine (R226G). Both mutants were fused to EGFP at their C-terminus and transformed into tobacco BY-2 cells (Fig. 6B). The predicted size of the fusion was 74 kDa. A band of expected size (74 kDa) could be detected upon western blot analysis using an antibody directed against GFP (Fig. 6C). However, in addition to the bona fide full-length protein, smaller bands could be detected by the anti-GFP antibody, presumably representing truncated versions of the protein. For the extract from cells expressing the wild-type VrMC5, two bands were observed, matching the predicted 52 and 47 kDa expected for cleavage at positions 139 and 226, respectively. Interestingly, the two mutated variants yielded only the larger band at 52 kDa, whilst the smaller band at 47 kDa was missing. Compared to the wild-type situation, also the band at 52 kDa was reduced in intensity. In the next step, we probed the response of these mutants to *cis*-3-hexenal. Without induction, mortality was low and similar to non-transformed cells, irrespective whether VrMC5 was mutated or not (Fig. 6D). Addition of *cis*-3-hexenal doubled mortality in the non-transformed cells but rose more than fourfold in cells expressing VrMC5. This increase was strongly quelled for the lines overexpressing the C139A and R226G mutants. When we analysed the auto-processing at 30 min (Fig. 6E), we found that for the VrMC5 line, the full-length band was clearly depleted in response to *cis*-3-hexenal as compared to the control, whilst the bands at 52 and 47 kDa had vanished, indicating that the protein had been auto-processed and the processing products subsequently consumed. For the mutant C139A, the full-length band was almost as abundant as in the control, and the 52 kDa band was still visible, although weaker than in the control, indicating that auto-processing had been slowed down considerably. In contrast, the R226G mutant showed a stronger depletion of the full-length band, but still a clearly visible band at 52 kDa, indicating that, here the auto-processing was moderately slowed down as compared to the non-mutated VrMC5 line.

### SA-dependent defence genes respond differently in auto-processing mutants of VrMC5

**Fig. 7:**
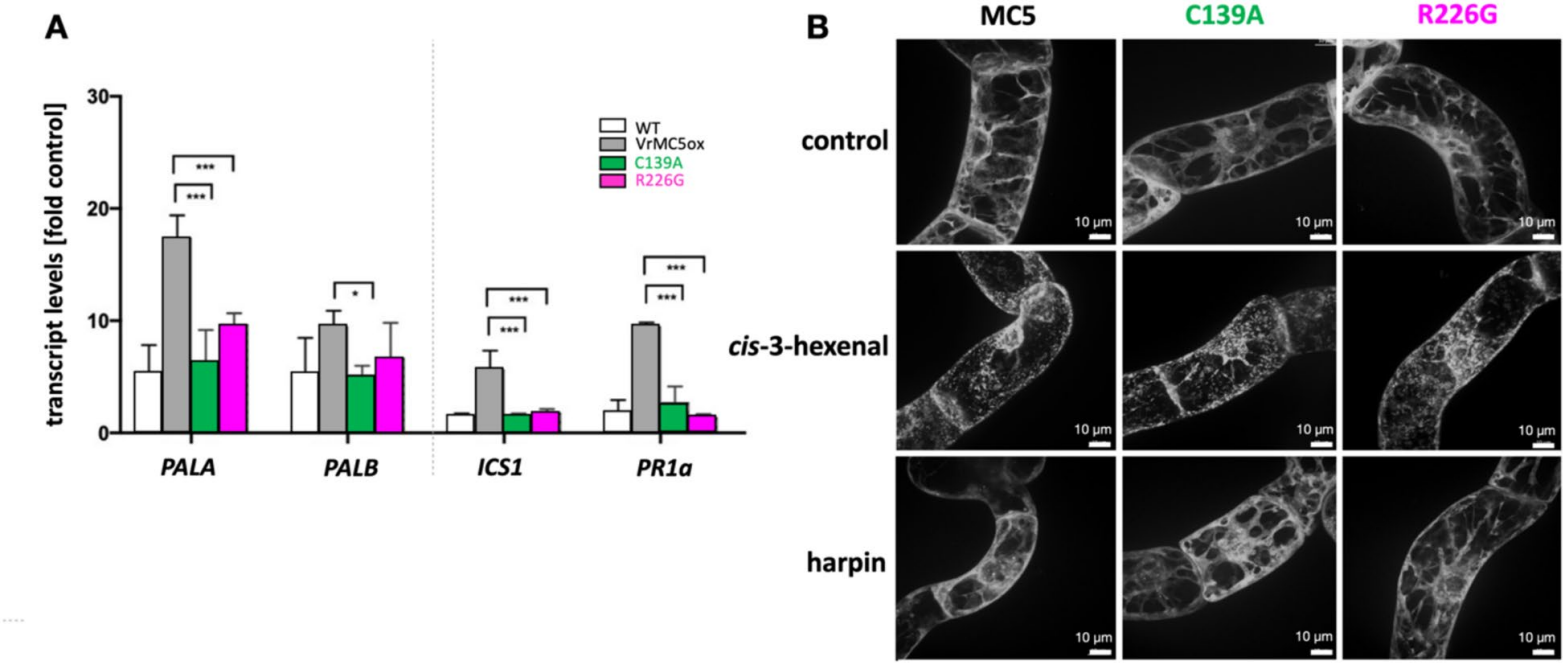
Auto-processing mutants of MC5 loose inducibility of SA-related transcripts by *cis*-3-hexenal although cytoplasmic remodelling is retained. **A** Steady-state transcript levels relative to the untreated control for *PHENYLAMMONIUM LYASE A* and B (*PALA* and *PALB*), *ISOCHORISMATE SYNTHASE 1* (*ICS1*), and *PATHOGENESIS-RELATED PROTEIN 1a* (*PR1a*) 30 min after addition of *cis*-3-hexenal (12.5 μM) in tobacco BY-2 cells expressing either the wild-type *VrMC5* or the two auto-processing mutants C139A and R226G. Transcript levels were estimated by real-time qPCR and normalised to EF-1α as the internal standard. Data represent the mean ± standard error (SE) of three independent biological replicates in technical triplicate, asterisks indicate significant differences with*P < 0.05, **P < 0.01 and ***P < 0.001 (Student’s *t *test). **B** The specific cytoplasmic remodelling is retained in the auto-processing mutants. Cells visualised at 60 min after addition of either *cis*-3-hexenal (12.5 μM) or 90 μg/ml harpin as specificity control

Whilst basal immunity is mostly dependent on jasmonates, HR is often associated with the activation of salicylic acid (SA) signalling (for review see Thaler et al. 2012). We asked, therefore, how the expression of SA-dependent defence genes, would be modulated upon overexpression of *VrMC5*, or processing mutants thereof. We observed significant amplification of the SA induction for the VrMC5 overexpressor for phenylammonium lyase *PAL1A* from fivefold to around 18-fold responses (Fig. 7A). Also, *PAL1B* was amplified but to a weaker extent. Also, *isochorismate synthase 1* (*IS1*) was amplified from around two-fold to six-fold, as well as *Pathogenesis-Related 1* (*PR1*), a well-known SA-responsive gene. Overexpression of the processing mutants did not yield this enhanced SA induction. Thus, processing *VrMC5* is necessary to amplify the response to SA.

Since *cis*-3-hexenal disintegrated the cytoplasmic signal for VrMC5-GFP (Fig. 4B), we wondered, whether this phenomenon would depend on auto-processing of VrMC5-GFP. Here the processing mutants of VrMC5 behaved in the same manner as the wild type by a disintegration of the cytoplasmic VrMC5-GFP signal (Fig. 7B, central row). Interestingly, the breakdown of trans-vacuolar strands, seen in the VrMC5-GFP wild-type line in response to harpin (Fig. 4B), was suppressed in the auto-processing mutants where cytoplasmic strands were retained (Fig. 7B, lower row), indicating that the role of auto-processing depends on the trigger (*cis*-3-hexenal versus harpin).

### Jasmonic acid mitigates *cis*-3-hexenal triggered mortality and gene expression

**Fig. 8:**
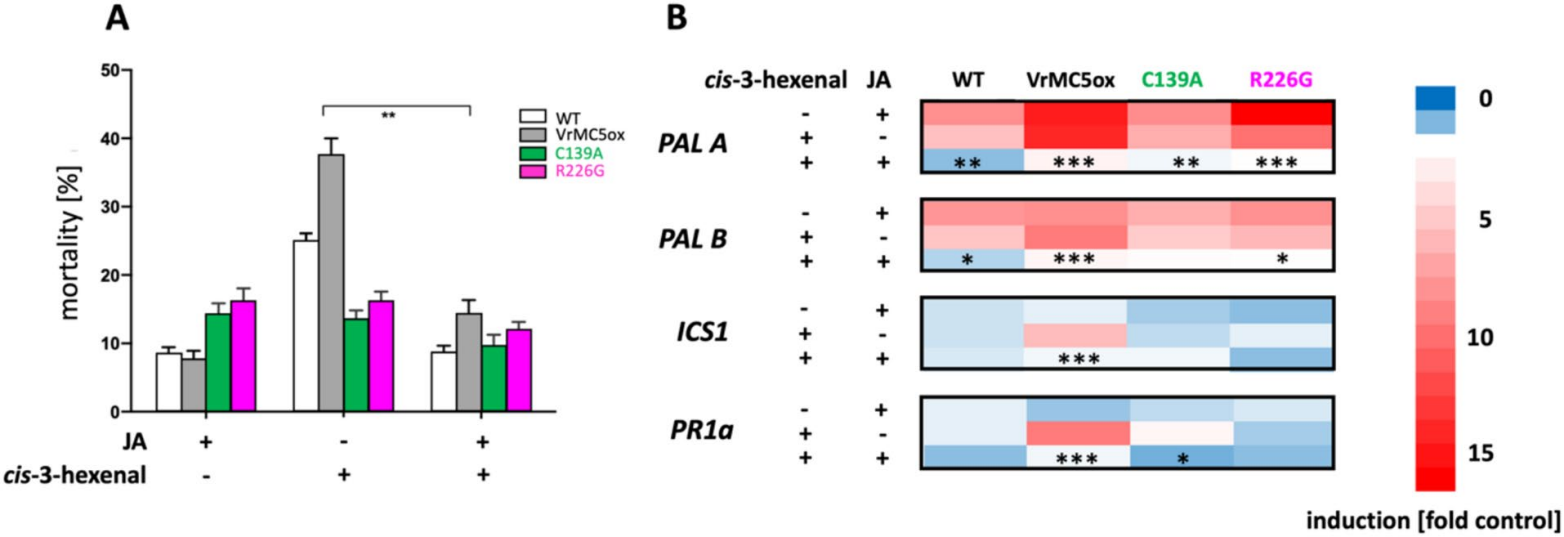
Pretreatment with jasmonic acid (JA, 100 μM, 30 min) mitigates mortality and inducibility of SA-related transcripts by *cis*-3-hexenal. **A** Mortality scored 30 min after addition of *cis*-3-hexenal (12.5 μM) in tobacco BY-2 cells expressing either the wild-type *VrMC5* or the two auto-processing mutants C139A and R226G. Data represent mean and standard errors from three independent experimental series counting 500 individual cells per data point and replication. **B** Steady-state transcript levels relative to the untreated control for *PHENYLAMMONIUM LYASE A* and B (*PALA* and *PALB*), *ISOCHORISMATE SYNTHASE 1* (*ICS1*), and *PATHOGENESIS-RELATED PROTEIN 1a* (*PR1a*) 30 min after addition of *cis*-3-hexenal (12.5 μM). Transcript levels were estimated by real-time qPCR and normalised to EF-1α as the internal standard. Data represent the mean from three independent biological replicates in technical triplicate, asterisks indicate significant differences between the induction by *cis*-3-hexenal with and without JA pre-treatment at significance levels *P* < 0.05 (*), *P* < 0.01 (**), and *P* < 0.001 (***) based on a Student’s *t *test)

To understand the functional context of *cis*-3-hexenal triggered mortality, we asked whether this phenomenon displays signatures of defence-related cell death (HR). Whilst HR depends on SA signalling (Thaler et al. 2012), basal immunity goes along with jasmonate signalling, antagonistic to SA (Chang et al. 2017). In fact, exogenous jasmonic acid can mitigate *cis*-3-hexenal triggered cell death (Akaberi et al. 2018). We tested, therefore, whether a pre-treatment with jasmonic acid would be able to quell the induction of SA biosynthesis and response genes by *cis*-3-hexenal, and how this would depend on auto-processing of VrMC5. Non-transformed BY-2 cells did not show any increased mortality in response to jasmonic acid (Fig. 8A), whilst 3-*cis*-hexenal more than doubled mortality. This mortality response was fully mitigated by pre-treatment with jasmonic acid. For the VrMC5-GFP strain, the induction of mortality by *cis*-3-hexenal was more pronounced. Again, a pre-treatment with jasmonic acid was able to suppress this mortality although completely. The two strains expressing the mutant versions of VrMC5-GFP responded to jasmonic acid by a higher mortality of ~ 15% over the non-transformed wild type and the VrMC5-GFP overexpressor (~ 8%) but failed to produce a response to *cis*-3-hexenal. Thus, pre-treatment with jasmonic acid was able to mitigate the mortality induced by *cis*-3-hexenal.

When we tested how this was mirrored in the expression SA-dependent genes (Fig. 8B), we found that jasmonic acid can induce transcripts for *PALA* in a similar manner as *cis*-3-hexenal. However, the combination of the two triggers did not add up, but instead silenced the induction. This was seen in non-transformed wild-type cells, and enhanced in amplitude, when *VrMC5-GFP* was overexpressed. The auto-processing mutants resembled the wild type rather than the *VrMC5-GFP* overexpressor. Also, the other tested genes (*PALB*, *ICS1*, *PR1*) showed this pattern albeit at a lower amplitude as compared to *PALA*. However, irrespective of the tested gene and the amplitude of the enhancement in the *VrMC5-GFP* overexpressor, jasmonic acid quelled the response to *cis*-3-hexenal strongly and significantly.

Thus, the mitigation of *cis*-3-hexenal induced mortality by jasmonic acid is reflected by a corresponding mitigation in the response of *cis*-3-hexenal-induced expression of genes related to salicylic acid synthesis and response.

### Proteolytic cleavage and enzymatic activity of VrMC5 in vitro depend on calcium

**Fig. 9:**
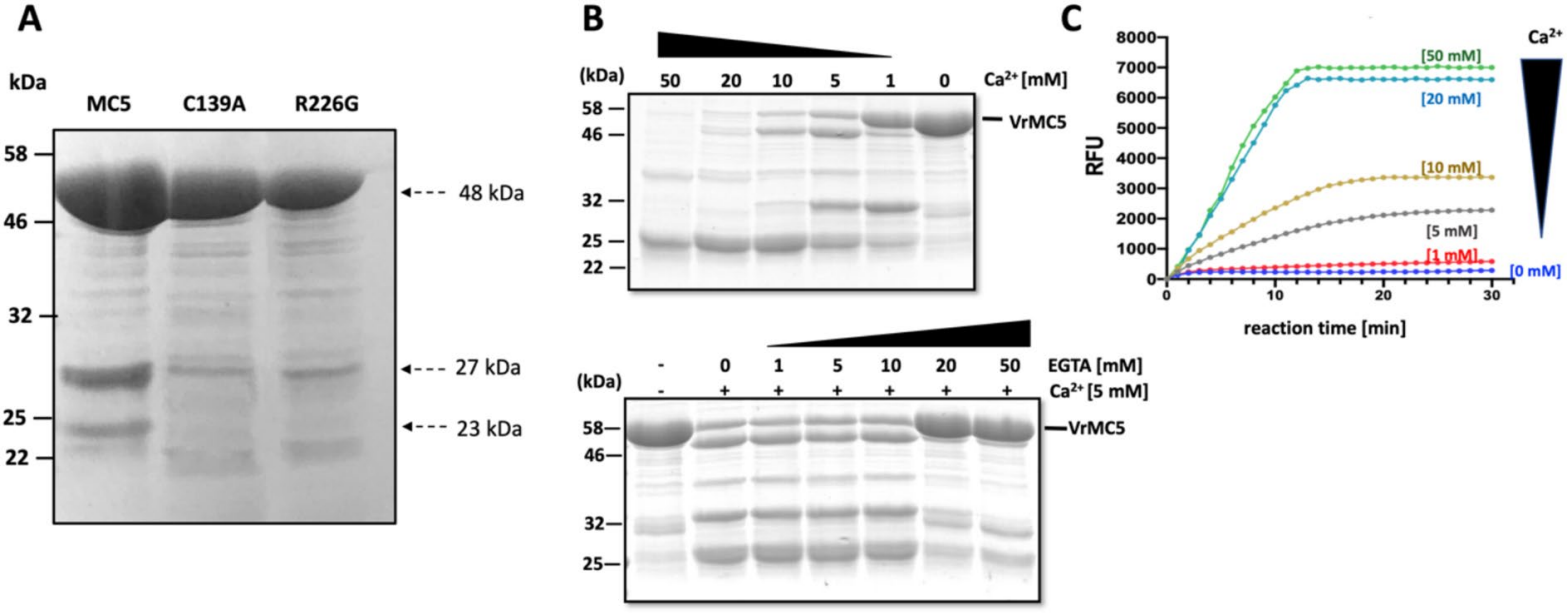
Autolysis and enzymatic activity of VrMC5 in vitro depend on calcium. **A** Recombinantly expressed VrMC5 and its mutants C139A and R226G separated by SDS-PAGE (12% acryl amide) upon visualisation with Coomassie Blue. 10 μg of protein loaded per lane. **B** VrMC5 processing in vitro over calcium concentration (upper image), and suppression of processing by adding the calcium chelator EGTA in the presence of 5 mM CaCl_2_ (lower image), both assessed after 20 min of incubation. **C** Dependence of enzymatic activity of recombinant VrMC5 on calcium in vitro measured by cleavage of the artificial substrate Boc-GRR-AMC releasing a fluorescent product upon cleavage of the tripeptide GRR after the Arg (R) residue that can be measured fluorometrically

Since auto-processing of type-II metacaspases depends on calcium (Watanabe and Lam 2011), we designed an *in vitro* assay based on recombinant expression of VrMC5 and its mutants. As this assay was exclusively based on biochemistry, we expressed VrMC5 alone, not fused to GFP. Following proteolysis by SDS-PAGE, staining by Coomassie Brilliant Blue. A strong band at around 48 kDa (Fig. 9A) corresponded to the expected size (46.7 kDa) of VrMC5, two fainter bands at 27 and 23 kDa matched the expected fragments upon cleavage at site 1, matching well the predicted molecular weight, and the two fragments excepted for cleavage at site 1 (Fig. 6A). Both side bands were depleted in the two mutants (C139A and R226G) of VrMC5 although their full-length proteins were well expressed. Upon incubation with calcium (Fig. 9B), the full-length protein disappeared when calcium increased, whilst the band at 27 kDa became stronger. When calcium exceeded 10 mM calcium, this 27 kDa band was progressively replaced by a 23 kDa band. The proteolytic decay could be completely suppressed by the calcium chelator EGTA (Fig. 9C), supporting that the supplemented calcium was the cause for the observed cleavage.

To find out whether this cleavage of VrMC5 activated enzyme activity, we used a commercially available fluorometric approach for type-II metacaspases (Vercammen et al. 2004), where metacaspases activity leads to release of the fluorescent reporter Amino-Trifluoromethyl-Coumarin (Fig. 9D). The reaction was started by supplementing the substrate Boc-GRR-AMC with calcium to different concentrations and then starting the reaction by adding 33 nM of purified VrMC5, recording the resulting fluorescence every minute for 30 min at a temperature of 20 °C as nmol of substrate hydrolysed per mg of protein and min (RFU). Up to 1 mM Ca^2+^, no significant activity was detected over the entire duration of this experiment. However, for 5 mM, fluorescence increased instantaneously and saturated at 30 min. For 10–20 mM, the amplitude increased, and the plateau was reached earlier. For 50 mM, saturation was reached for both effects. Thus, enzyme activity depends on calcium in a roughly sigmoidal dose–response with a dynamic range between 5 and 20 mM, matching well with the dynamic range for proteolysis (compare Figs. 9B and C). Thus, the stimulation of VrMC5 cleavage by calcium is tightly correlated with the stimulation of enzymatic activity for this metacaspases.

### Autolysis and enzymatic activity of VrMC5 in vitro depend on Cys139 and Arg226

**Fig. 10:**
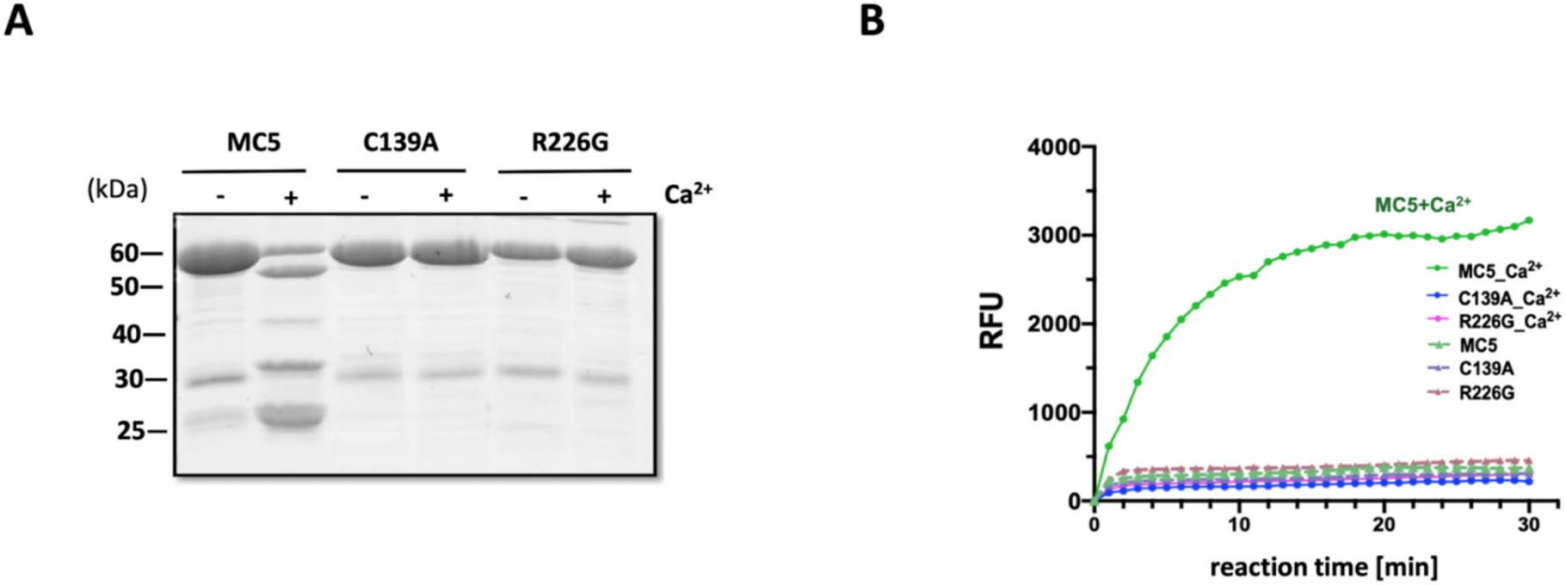
Self-processing and enzymatic activity of VrMC5 depend on Cys139 and Arg226. **A** Autolysis of VrMC5 WT and its mutants in vitro after incubation with 5 mM CaCl2. Mixtures were separated on 12% SDS-PAGE and bands were visualised by Coomassie Blue staining. **B** Enzymatic activity of recombinant VrMC5 and its mutants in vitro either in the absence or the presence of 10 mM CaCl_2_. Measured by cleavage of the artificial substrate Boc-GRR-AMC releasing a fluorescent product upon cleavage of the tripeptide GRR after the Arg (R) residue that can be measured fluorometrically. Purified proteins were added at 30–40 nM to the assay. C139A, purified VrMC5C139A; R226G, purified VrMC5R226G

The proteolytic decay of VrMC5 is depending on calcium as is the activation of metacaspase activity measured in the fluorometric assay. If this proteolysis is expression of auto-processing, we would predict that it is impaired in the two mutants of VrMC5. When we exposed recombinant VrMC5 along with its mutants C139A and R226G to calcium, we did not observe any stimulation of proteolytic decay in those mutants, contrasting with the breakdown of the wild-type protein (Fig. 10A). When we tested the enzymatic activity of the mutant proteins, there was none to be detected, no matter, whether calcium was added or not (Fig. 10B). This was, again, contrasting with the behaviour of the wild-type protein, whose enzymatic activity was strongly stimulated by calcium in the same assay. This strict dependence of proteolytic decay and enzyme activation on Cys139 and Arg226 strongly supports the specificity of both phenomena and also meets all criteria posed to a true auto-processing of VrMC5.

## Discussion

In the current work, we investigated the link between the class-II metacaspases VrMC5 for *Vitis rupestris*, a North American wild grapevine species. A cell culture derived from this species is able to deploy a hypersensitive response in response to biotrophic pathogens, bacterial elicitors, and the volatile defence compound 3-*cis*-hexenal. Using tobacco BY-2 cells expressing GFP fusions of VrMC5, we find an association with nucleus-associated microtubule arrays. We then probe the functional context of this microtubule association using *cis*-3-hexenal, a trigger, which cannot only deploy cell death but also activate the expression of defence genes. Cytoskeletal breakdown precedes and is required for cell death in response to *cis*-3-hexenal. This cell death response as well as the accompanying gene expression is mitigated by jasmonic acid, coming from a metabolic pathway that is concurrent with the synthesis of *cis*-3-hexenal by competing for the precursor 13(S)-hydroperoxylinolenic acid (13-HPOT). Using two mutants of VrMC5, where two crucial sites have been mutated, we show that these sites are required for calcium activation of metacaspases activity and auto-processing in vitro. Upon expression of these mutants as GFP fusions in tobacco cells, we show further that these sites are also needed for cell death and expression of defence genes in response to *cis*-3-hexenal.

These findings lead to the following questions: (1) What is the role of microtubules in defence-related cell death? (2) What is the functional context for the mitigation of defence-related cell death by jasmonates? (3) How do microtubules participate in the cellular decision to deploy defence-related cell death?

### Are microtubules keeping metacaspases on the leash?

Metacaspase 5 is associated with nucleus-associated microtubules (Figs. 1, 2) but not with cortical microtubules (Suppl. Fig. S1). The decoration of microtubule arrays found in cycling cells might be a mechanism to ensure symmetric distribution of metacaspases to the daughters. Alternatively, the association with microtubules might negatively regulate the auto-processing of metacaspases 5. Using *cis*-3-hexenal as trigger, we observe an early breakdown of microtubules (Fig. 5A), and when we stabilise microtubules by taxol, this mitigates the mortality in response to *cis*-3-hexenal (Fig. 5B). The association with microtubules is, therefore, relevant to the function of metacaspases, precedes and is required for cell death in response to *cis*-3-hexenal. The notion that interaction with microtubules bears on the metacaspase function is also supported by the finding from EPC-affinity chromatography, where metacaspase 5 associated with the de-tyrosinated subpopulation of α-tubulin is de-tyrosinated (Fig. 3B). Detyrosinated α-tubulin is not only a marker for stable microtubules, but also binds to phospholipase D, a central hub for stress signalling (Zhang et al. 2022).

These findings add a further facet to the link between microtubules and defence. Not only are microtubules remodelled during defence against various pathogens (for a classical review, see Hardham 2013), but also targeted by bacterial effectors that bind to and modify microtubules (Huei Yi et al. 2012), or alter the binding of microtubule-associated proteins such as kinesin motors (Lee et al. 2019) or MAP65 (Guo et al. 2016). Since microtubules are targets for bacterial effectors, they might be required to execute cell death-related immunity. Association of autophagosome components such as ATG8 from *A. thaliana* (Ketelaar et al. 2004), or the autophagy receptor Joka2 with microtubules (Zientara-Rytter and Sirko 2014) indicates that microtubules are physically linked with the machinery that executes cell death. There seems to be a functional association as well since disruption of microtubules can trigger chloroplast autophagy (Wang et al. 2015). Likewise, kinetin-induced cell death in tobacco cells is preceded by a specific breakdown of microtubules (Kaźmierczak et al. 2023). The link between microtubules and autolysis might be evolutionarily ancient since it is well-known from animal cells (for review, see Mackeh et al*.* 2013). Interestingly, a motif highly conserved amongst type-II metacaspases (including AtMC4) shows more than 90% similarity with the microtubule-binding domain of the human MAP1 A/B light-chain 3B (Fig. 3A, inset) already identified in the autophagy gene AtATG8i (Gardiner and Marc 2003).

Therefore, the working model that microtubules keep VrMC5 on the leash is supported by decoration of microtubules in vivo, binding to tubulin in vitro, mitigation of function by taxol, and the presence of a microtubule-binding motif in a highly conserved region of type-II metacaspases shared with other proteins involved in autolysis.

### What is the functional relevance of metacaspase mitigation by jasmonates?

A rapid activation of cell death and the rapid induction of salicylic acid-related transcripts are salient responses to *cis*-3-hexenal (Fig. 8). Both responses can be suppressed by jasmonic acid. This pattern is congruent with previous observations in the same cell strain, tobacco BY-2, where the hypersensitive response evoked by harpin, an elicitor from a phytopathogenic bacteria, was mitigated in the presence of jasmonate (Akaberi et al. 2018). Along the same line, a comparison of basal immunity elicited by the bacterial elicitor flg22 and the hypersensitive response triggered by harpin in grapevine cells (Chang et al. 2017) revealed that jasmonate and its bio-active isoleucine conjugate accumulated exclusively in response to flg22, i.e. in the context of basal immunity, whilst the hypersensitive response induced by harpin did not lead to jasmonate accumulation. Jasmonates are central for the wounding response and the defence against necrotrophic pathogens, where a hypersensitive response is not a successful strategy. In contrast, salicylic acid is associated with the defence against biotrophic pathogens, where programmed cell death is a very efficient strategy to ward off the intruder. This divergence of defence responses is accompanied by a general antagonism in the activity of jasmonate and salicylic acid, which has been reported for almost twenty plant species from quite different taxa and, therefore, must have been acquired early in the evolution of seed plants (Thaler et al. 2012).

It seems that the divergence in the response is linked with a bifurcation early in the processing of the stress signal: The CYP74 enzymes processing the 13-hydroperoxides formed during stress-triggered membrane degradation have been duplicated early in the evolution of land plants (Lee et al. 2008): The CYP74A enzymes (known as Allene Oxide Synthases, AOS) initiate the synthesis of the life-saving jasmonates. Their twins from the CYP74B clade transform the same substrate into the volatile aldehydes *cis*-3-hexenal and 2-*trans*-hexenal. Whilst 2-*trans*-hexenal seems biologically inactive, its isomer, *cis*-3-hexenal efficiently triggers actin remodelling and cell death (Akaberi et al. 2018). In other words, a metabolic divergence in signal genesis represents an early decision between life and death. This early decision is subsequently amplified by antagonistic effects of jasmonate and salicylate signalling. For instance, the master regulator of salicylic acid-induced gene expression, NONEXPRESSER OF PR GENES1 (NPR1), can bind and block MYC2, the master regulator of jasmonate-induced gene expression (Nomoto et al. 2021). On the other hand, there exist several cases, where stimulation of MYC2 activity by jasmonates leads to suppression of salicylic acid responses (for review see Thaler et al. 2012). Of particular interest is here the suppression of hypersensitive cell death by the bacterial effector coronatine, a highly potent mimic of bio-active jasmonates in the interaction of *Pseudomonas syringae* with *A. thaliana* (Brooks et al. 2005).

Our findings are not only consistent with this reported role of jasmonate in the mitigation of defence-related cell death, but extend these findings. Since this mitigation is retained in cells overexpressing MC5, both with respect to cell death and to salicylate-related gene expression, jasmonates are not acting upstream of type-II metacaspase activation (in our experiment triggered by *cis*-3-hexenal). There is an interesting detail here: PAL transcripts are induced by *cis*-3-hexenal in a comparable manner between wild type and VrMC5 overexpressor, which is suppressed by jasmonate in both cases (Fig. 8B). In contrast, the salicylate synthesis gene *ICS1* and the salicylate response gene *PR1* are induced depending on the overexpression of VrMC5, which is suppressed by jasmonate. Here, jasmonate seems to interfere downstream of type-II metacaspases. Whether it targets class-I metacaspases or other autolytic enzymes controlled by type-II metacaspases represents an interesting question for future research.

### How do microtubules participate in the cellular decision to deploy defence-related cell death?

Whilst microtubules seem to interact with metacaspases, and whilst microtubular stabilisation seems to interfere with metacaspase activity, it is not clear how microtubule integrity might be connected to the phytohormonal signalling regulating the execution of cell death. In this context, heat shock protein 90 (HSP90) is of interest. This protein is shared in protein complexes regulating both, jasmonate and salicylic acid, signalling (for review, see di Donato and Geisler 2019). As a binding partner of SGT1b and HSP70, HSP90 binds and stimulates COI1, a crucial component of the jasmonate receptor complex (Zhang et al. 2015). In contrast, by interaction with the transcription factor WRKY20, HSP90 can promote the synthesis of salicylic acid (Wei et al. 2021).

However, HSP90 is not only a binding partner recruited by concurrent signalling complexes, it is, also, a microtubule-binding protein in both, animals (Weis et al. 2010; Lange et al. 2000) and plants (Freudenreich and Nick 1998; Petrásek et al*.* 1998; Krtková et al. 2012). Thus, the cellular decision for or against cell death converges on two levels on microtubules. First, the decision between jasmonate versus salicylic acid signalling depends on the availability of HSP90, which can be tethered on microtubules. Second, the release of type-II metacaspases as regulators of programmed cell death depends on their interaction with microtubules. It will be interesting to test in the future, whether there is a functional link between the two decisions, and whether this link can be manipulated by targeting HSP90.

### Outlook

The current work develops the working hypothesis that microtubules participate in the regulation of cell death, probably by tethering type-II metacaspases. A testable implication of this hypothesis would be that inhibition of HSP90 activity, for instance by Geldanamycin, should modulate cell death, for instance in response to *cis*-3-hexenal. Using EPC-affinity chromatography in concert with proteomics, it should be possible to identify binding partners mediating between HSP90 and MC5. Furthermore, it would be rewarding to explore whether metacaspase sequestration is also used to restrain other forms of PCD. For instance, a microtubular breakdown has also been observed for the cell death response to cytokinin kinetin (Kaźmierczak et al. 2023) indicating a scenario, where the microtubule–metacaspase module might be a common theme in different types of signal-dependent cell death.

## Supplementary Information

Below is the link to the electronic supplementary material.

Supplementary file1 (xlsx 421 KB)

## Data Availability

The data supporting the findings of this study are available from the corresponding author upon request.
